# Impact of gold nanoparticle size and coating on radiosensitization and generation of reactive oxygen species in cancer therapy†

**DOI:** 10.1039/d4na00773e

**Published:** 2025-01-09

**Authors:** E. Loscertales, R. López-Méndez, J. Mateo, L. M. Fraile, J. M. Udias, A. Espinosa, S. España

**Affiliations:** a Grupo de Física Nuclear, EMFTEL & IPARCOS, Universidad Complutense de Madrid Pl. de las Ciencias, 1, Moncloa-Aravaca Madrid Spain esloscer@ucm.es lmfraile@ucm.es jmudiasm@ucm.es sespana@csic.es; b Instituto de Tecnologías Físicas y de la Información “Leonardo Torres Quevedo” (ITEFI-CSIC) C/Serrano, 144 Madrid Spain; c IMDEA Nanociencia C. Faraday, 9, Fuencarral-El Pardo Madrid Spain rosalia.lopez@imdea.org; d Centro Nacional de Investigaciones Cardiovasculares (CNIC) C. de Melchor Fernández Almagro, 3, Fuencarral-El Pardo Madrid Spain; e Instituto de Investigación del Hospital Clínico San Carlos (IdISSC) C. del Prof Martín lagos, S/N, Moncloa-Aravaca Madrid Spain; f Instituto de Ciencia de Materiales de Madrid (ICMM-CSIC) Campus de Cantoblanco, C. Sor Juana Iés de la Cruz, 3, Fuencarral-El Pardo Madrid Spain ana.espinosa@csic.es; g Unidad de Nanomateriales Avanzados, IMDEA Nanociencia, Unidad Asociada al CSIC por el ICMM-CSIC c/Faraday 9 28049 Madrid Spain

## Abstract

Radiation therapy is a common cancer treatment but often damages surrounding healthy tissues, leading to unwanted side effects. Despite technological advancements aimed at improving targeting, minimizing exposure to normal cells remains a major challenge. High-Z nanoparticles, such as gold nanoparticles (AuNPs), are being explored as nano-radiosensitizers to enhance cancer treatment through physical, biological, and chemical mechanisms. This study focuses on evaluating the chemical and biological radiosensitizing effects of AuNPs exposed to ionizing radiation (0–50 Gy), specifically their production of reactive oxygen species (ROS) and their impact on cancer cells. ROS generated by AuNPs of varying sizes and coatings were quantified using fluorescence probes for hydroxyl radicals (HO·) and singlet oxygen (^1^O_2_). The radiosensitizing effects on MDA-MB-231 cancer cells were assessed *via* clonogenic assays. Our results show a clear dependence of ROS production on AuNP size. Interestingly, PEG-capped AuNPs did not significantly enhance HO· production but greatly increased ^1^O_2_ production, suggesting that multiple reactive species contribute to the radiosensitization process. Clonogenic assays confirmed that PEG-capped AuNPs produced stronger radiosensitizing effects than citrate-capped AuNPs, with smaller AuNPs providing more pronounced biological effects. This study underscores the importance of conducting both chemical and biological evaluations to fully understand the radiosensitization efficacy of AuNPs.

## Abstract 2

Radiosensitization by AuNPs depends on size and coating, with PEG-capped nanoparticles increasing ^1^O_2_ production and surpassing citrate-capped counterparts, especially for smaller particles.

## Graphical abstract

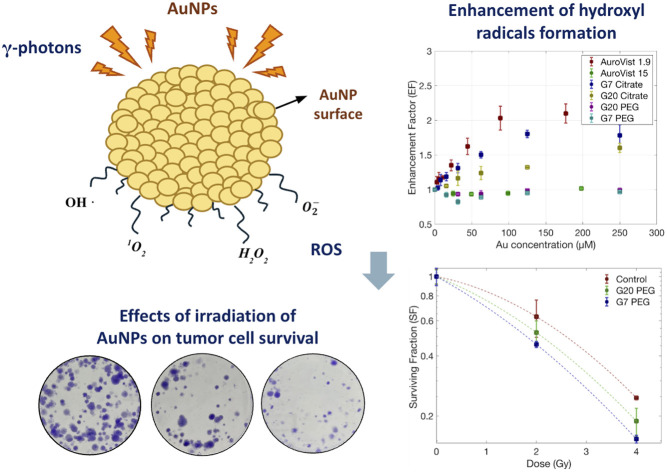

## 1. Introduction

A significant number of cancer patients undergo radiation therapy^1–3^ during their treatment either alone or in combination with surgery or chemotherapy. However, a major limitation is the potential damage induced to surrounding healthy tissues, which can cause side effects. While advancements in technology, planning, and imaging guidance have improved the ability to spare healthy tissue, the risk of both acute and late toxicities remains a key limiting factor. Therefore, one of the main challenges in radiation therapy is to maximize the radiation dose to cancer cells, while minimizing exposure to normal cells.

Nano-radiosensitizers^4^ like high-Z nanoparticles^5–8^ have been proposed to enhance cancer therapies *via* three principal mechanisms: physical, biological, and chemical enhancement. High-Z materials exhibit higher attenuation cross-section absorbing more energy compared to water at the tumor site and therefore, resulting in an enhanced damage on cancer cells.^9,10^ Biological effects^11^ involve the role of nano-radiosensitizers in inducing oxidative stress, modulating the cell cycle, and causing bystander effects.^12^ Additionally, the surface atoms can act as a catalytic platform^13^ which is increased with size reduction of these nanoparticles.^13–15^ However, increased stability requires dense coating or functionalization of nano-radiosensitizers^16,17^ which can restrict the chemical enhancement effect.^18^

While numerous studies have investigated the physical, chemical, and biological effects^19–23^ of nano-radiosensitizers separately, additional research integrating these aspects is essential to improve our understanding of their combined impact on cancer treatment. Comprehensive studies that simultaneously examine these mechanisms could provide a complete view of how nano-radiosensitizers interact within the biological environment, potentially uncovering synergistic effects that are not evident when each mechanism is considered in isolation. By bridging the knowledge gap between these interconnected effects, we can develop more effective and optimized cancer therapies that fully exploit the potential of high-Z nanomaterials.

In this study, we evaluated both chemical and biological radiosensitizing effects of gold nanoparticles (AuNPs). We used AuNPs with different sizes (from 1.9 to 20 nm) and coatings (citrate and PEG), including both commercially available and synthesized nanoparticles. To gain a more comprehensive understanding of the effect of different nanoparticle configurations on the chemical environment, we quantified the production of ROS species: HO· and ^1^O_2_ using fluorescence probes such as coumarin and singlet oxygen sensor green (SOSG). Coumarin and SOSG offer low background fluorescence, strong signal intensity, and easy use in biological systems. SOSG is particularly valuable for detecting and measuring ^1^O_2_, which is difficult to track due to its short lifetime. We then assessed the radiosensitizing effects of the same AuNPs on cancer cells to evaluate their biological and therapeutic impact.

## 2. Methods & materials

### 2.1. Materials

Coumarin-3-carboxylic acid 99% (C_10_H_6_O_4_, coumarin), tri-sodium citrate dihydrate (Na_3_C_6_H_5_O_7_·2H_2_O), gold(iii) chloride hydrate (HAuCl_4_·H_2_O), sodium borohydride (NaBH_4_), thiolated PEG (Thiol-PEG, 2000 MW) and 2′,7′-dichlorofluorescin diacetate (DCFH-DA) were purchased from Sigma-Aldrich. Singlet Oxygen Sensor Green (SOSG) was purchased from Thermo Fisher Scientific (USA). Gold nanoparticles, AuroVist 1.9 nm and AuroVist 15 nm, were obtained from Nanoprobes (USA). All other chemicals, unless mentioned, were purchased from Sigma-Aldrich (USA).

### 2.2 Synthesis AuNPs

#### 2.2.1 Citrate-capped 20 nm AuNPs (G20)

Citrate-capped 20 nm AuNPs were synthesized following the Turkevich method.^24^ First, 38 mL of distilled water was mixed with 1 mL of 10 mM HAuCl_4_ and the solution was brought to ebullition. Then, 1 mL solution of tri-sodium citrate dihydrate was added while being vigorously stirred obtaining a final citrate concentration of 2.5 mM. The heating and the stirring were maintained for several minutes and aliquots were collected from the solution at different reaction times in order to monitor nanoparticle growth by recording their optical density at 520 nm (OD@520) (see Fig. S1†). Once the growth process is completed, the OD@520 reaches a plateau and the solution is left to cool down to room temperature (RT). Finally, in order to remove residual reactants, the AuNPs were washed with distilled water by 3 centrifugation steps at 3000×*g* for 45 min each.

#### 2.2.2 Citrate-capped 7 nm AuNPs (G7)

Citrate-capped 7 nm AuNPs were synthesized following the method described at Cheng *et al.*^14^ First, 10 mL of 10 mM HAuCl_4_ was mixed with 10 mL solution of 20 mM tri-sodium citrate dihydrate. The solution was vigorously stirred for 5 minutes at RT and afterwards, 20 mL of a 2.2 mM sodium borohydride solution was added. The stirring was maintained for 45 minutes. Finally, in order to remove residual reactants, the AuNPs were washed with distilled water by 3 centrifugation steps at 9000×*g* for 10 min each, using 30 kDa Amicon ultra-centrifugal filters (Millipore, MA, USA).

#### 2.2.3 Ligand-exchange

Both G20 and G7 PEG-coated AuNPs were obtained by adding an excess of thiol-PEG to citrate capped AuNPs with final concentrations of 1 mM Au and 0.025 mM thiol-PEG and stirring for 1 hour at RT (Fig. S2†). Resulting PEG-coated AuNPs were washed with distilled water using the same protocol as for the corresponding citrated-capped AuNPs. Stability of PEG-coated AuNPs was studied by diluting them in PBS and monitoring their OD@520 for 24 hours (see Fig. S2†). All synthesized nanoparticles were stored at 4 °C in order to prevent aggregation.

### 2.3. Morphological and elemental characterization of AuNPs

The size and morphology of AuNPs was observed by transmission electron microscopy (TEM) using a JEOL JEM-2100 microscope. Samples were prepared by placing a drop of suspension into a 200-mesh carbon-coated copper grid and were allowed to air dry before being inserted into the microscope. Size measurements were performed on ImageJ software. Hydrodynamic size and zeta (*ζ*) potential were measured using a Zetasizer Nano ZS device (Malvern Panalytical Instruments, UK) with a laser at 633 nm and an angle of 173° between the detector and the sample. Gold concentration analysis was performed by inductively coupled plasma mass spectrometry (ICP-MS). For that purpose, 100 μL of AuNPs were digested by addition of 0.15 mL of aqua regia (a 1 : 3 mixture of nitric acid (68%) and hydrochloric acid (37%)) and the mixture was incubated at 50 °C for 5 days. The samples were then analyzed using ICP-MS to measure the Au concentration.

### 2.4. Irradiation with γ photons

A γ-rays irradiator with a Cesium-137 gamma source was used to irradiate samples in this study. Samples for ROS quantification were irradiated at a dose rate of 6 Gy min^−1^ in a 0–50 Gy dose range, in PCR strips and transferred to 96-well plates after irradiation for further analysis. The dose range for each specific ROS sensor was optimized according to the dynamic range of the fluorescent signal. Cells were irradiated at a dose rate of 0.56 Gy min^−1^ in a 0–4 Gy dose range. In this case cells were incubated in 96-well plates and transferred to 6-well plates after irradiation to perform clonogenic assays.

### 2.5. ROS quantification

The production of HO· was quantified using a coumarin-based compound, a probe that generates highly fluorescent products upon interaction with HO·, being the major fluorescent product 7-hydroxycoumarin-3-carboxylic acid (7-OHCCA). For that purpose, samples were prepared containing a solution of 0.5 mM coumarin in distilled water with different AuNP concentrations ranging from 0 to 300 μM Au and irradiated with γ photons at doses of 10 and 25 Gy. After irradiation, pH of samples was adjusted with 0.02 M phosphate buffer (pH = 6.8). Next, the fluorescence intensity of samples was measured using a Victor Nivo Microplate Reader (PerkinElmer). The excitation and emission filters were set at 315–395 nm and 430–490 nm, respectively. Measured values were corrected for the light attenuation produced by AuNPs as explained in the next section.

The production of ^1^O_2_ was monitored using SOSG,^25^ a highly specific fluorescence probe for the detection of ^1^O_2_. In this case, samples were prepared containing a solution of 0.1 μM SOSG in distilled water with different AuNPs concentrations ranging from 0 to 25 μM Au and irradiated with γ photons at doses of 5 and 10 Gy. After irradiation, pH of samples was adjusted by diluting them in PBS (pH = 7.4). Next, the fluorescence intensity of samples was measured setting the excitation and emission filters at 450–510 nm and 500–560 nm, respectively. In this case, attenuation correction was not needed because it was negligible at the concentrations used. Independent experiments were performed in triplicate for each sample.

The relative production of HO· and ^1^O_2_ radicals were quantified as the slope of a linear fit between the recorded fluorescence values and the radiation dose for each concentration and type of AuNP. The enhancement factor (EF) was defined as the ratio between the slopes obtained for samples containing AuNPs and control samples.

#### 2.5.1 Attenuation correction

The attenuation correction for coumarin measurements was performed by comparing the fluorescence of irradiated and non-irradiated coumarin mixed with either water or AuNPs. The following steps were taken to accomplish this correction. A vial was filled with 10 mL of coumarin (1 mM) and irradiated with a dose of 50 Gy. Next, irradiated and non irradiated coumarin was diluted by half with AuNPs or distilled water and fluorescence values were measured. The values obtained for AuNPs and water samples were fitted to a straight line for each AuNP and Au concentration. Attenuation correction was performed using the corresponding linear fit (Fig. S3†). Each linear fit was performed using only two points. However, linearity was previously verified using multiple points with mixtures of different concentrations of irradiated and non-irradiated coumarin.

### 2.6. *In vitro* studies

*In vitro* studies were performed on MDA-MB-231 breast cancer cells in order to assess cellular uptake, toxicity and radiosensitization effects when incubated with AuNPs. MDA-MB-231 cells were cultured in Dulbecco's modified Eagle's medium (DMEM) supplemented with 10% fetal bovine serum and 1% penicillin/streptomycin and maintained in an incubator at 37 °C and 5% CO_2_ under humid conditions.

#### 2.6.1 Cell internalization

To investigate cellular internalization of AuNPs,^26^ 300 000 cells per well were seeded in 6-well plates and allowed to adhere. Afterwards, cells were incubated for 24 hours with AuNPs at a concentration of 200 μM of Au. Then, cells were washed three times with PBS and trypsinized for cell counting. Subsequently, the samples were sonicated for 1 hour at 60 °C, centrifuged at 9000×*g* for 5 minutes and the supernatant was removed to isolate the pellet. The pellet was digested for 5 days in 0.15 mL of aqua regia at 50 °C. Next, samples were analyzed by ICP-MS and the total Au mass of each sample was determined. AuNP internalization was obtained as the ratio of the total Au mass and the number of cells.

#### 2.6.2 Radiosensitization effect

In order to quantify the radiosensitization effect of AuNPs, clonogenic assays were performed as follows. 20 000 cells per well were seeded in 96-well plates and incubated for 24 hours with AuNPs at extracellular concentration of 1 mM Au. Then, cells were washed with PBS and fresh medium was added. Cells were irradiated with γ photons with radiation doses including 0, 2 and 4 Gy. After irradiation, cells were trypsinized, counted and seeded in 6-well plates including 300 cells per well for 0 and 2 Gy and 900 cells per well for 4 Gy. Cells were incubated for 16 days to form colonies. Colonies were fixed with formaldehyde and stained with 0.5% crystal violet. Pictures were taken and colonies were counted manually with ImageJ. Each condition was replicated three times. Surviving fractions (SF) were calculated relative to non irradiated control cells and the cell survival curves were fitted using the linear quadratic model (LQ). The LQ model describes the relationship between the cell survival and radiation dose as shown in eqn (1):

1 SF = exp^(−*αD*−*βD*^2^)^

where *α* and *β* are the linear and quadratic parameters and *D* is the absorbed dose. We also evaluated the surviving fraction at 4 Gy (SF_4_), the *α*/*β* ratio, and the sensitizer enhancement ratio (SER) of each AuNP. SER was calculated as the ratio of the mean inactivation dose (MID) obtained for control and AuNP-incubated cells.^27^ MID was obtained as the area under the surviving fraction curve.

All experiments were carried out in triplicate. Statistically significant differences between control and AuNP-incubated cells were calculated using the two-tailed unpaired *t*-test or one-way analysis of variance with a *p* value of <0.05 considered significant.

#### 2.6.3 Cytotoxicity

In order to determine AuNP toxicity, the colony forming ability on non irradiated cells incubated with AuNPs was measured. Toxicity results were acquired from the results obtained for non-irradiated cells in cell survival clonogenic assays. The number of colonies counted for each AuNP was compared to the number of colonies formed for control cells.

#### 2.6.4 Intracellular ROS quantification

The intracellular ROS production was quantified following the next protocol. 20 000 cells per well were seeded in 96-well strip-well plates and incubated for 24 hours with AuNPs at a extracellular concentration of 1 mM Au. Then cells were washed with PBS and incubated for 30 min with 100 μM of DCFH-DA diluted in PBS. Next, cells were washed with PBS and irradiated with γ photons with radiation doses including 0, 5 and 10 Gy while they were covered with PBS. After irradiation, fluorescence intensity of samples was measured using a Victor Nivo Microplate Reader (PerkinElmer). The excitation and emission filters were set at 450–510 nm and 500–560 nm, respectively. All steps including DCFH-DA were carried out in the dark under light conditions studied previously which do not trigger the photo-activation of the sensor. The slope of a linear fit between the recorded fluorescence values and the radiation dose was obtained for each type of AuNP and the enhancement factor (EF) was obtained as the ratio between the slopes obtained for samples containing AuNPs and control samples.

## 3. Results

### 3.1. AuNPs synthesis and characterization

**Fig. 1:**
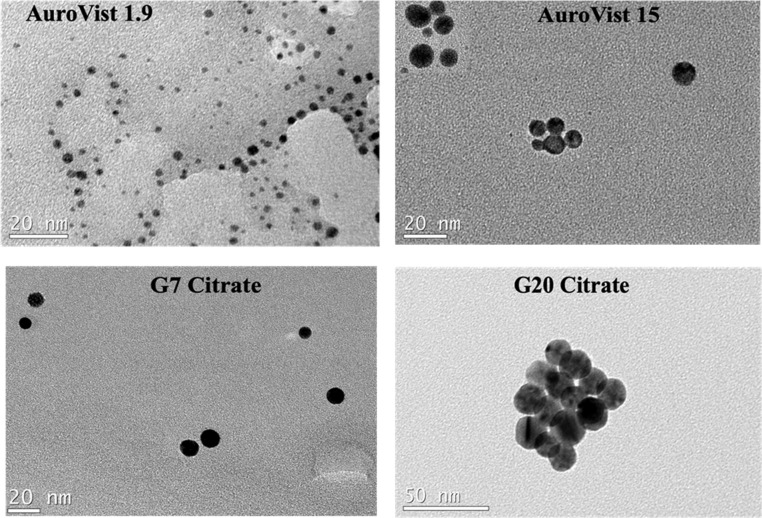
TEM images of some of the AuNPs used in this study.

Commercial (AuvoVist 1.9 and 15 nm) and synthesized (citrate and PEG coated G20 and G7) AuNPs were examined by transmission electron microscopy (TEM) for shape (spherical or slightly ovoid) and size measurements (see Fig. 1). The main characteristics of the AuNPs are summarized in Table 1. The size was obtained as the mean and standard deviation of at least 30 measurements.

**Table 1:** Main characteristics of the AuNPs used in this study, including the particle coating and the size measured by TEM

AuNP	Size (nm)	Hydrodynamic size (nm)	Surface charge (mV)	Coating
AuroVist 1.9	1.9 ± 0.3	3.9 ± 0.1	−8.4 ± 2.1	Unknown
AuroVist 15	10.9 ± 0.9	52.9 ± 0.2	−19.9 ± 1.3	Unknown
G20 citrate	19.6 ± 1.6	36.7 ± 0.7	−17.9 ± 1.0	Citrate
G7 citrate	7.5 ± 1.9	24.2 ± 0.1	−24.2 ± 0.6	Citrate
G20 PEG	20.1 ± 1.2	34 ± 0.1	−16.7 ± 0.9	PEG
G7 PEG	7.6 ± 0.9			PEG

### 3.2. Radio-activated production of hydroxyl radicals (HO·) in the presence of AuNPs

**Fig. 2:**
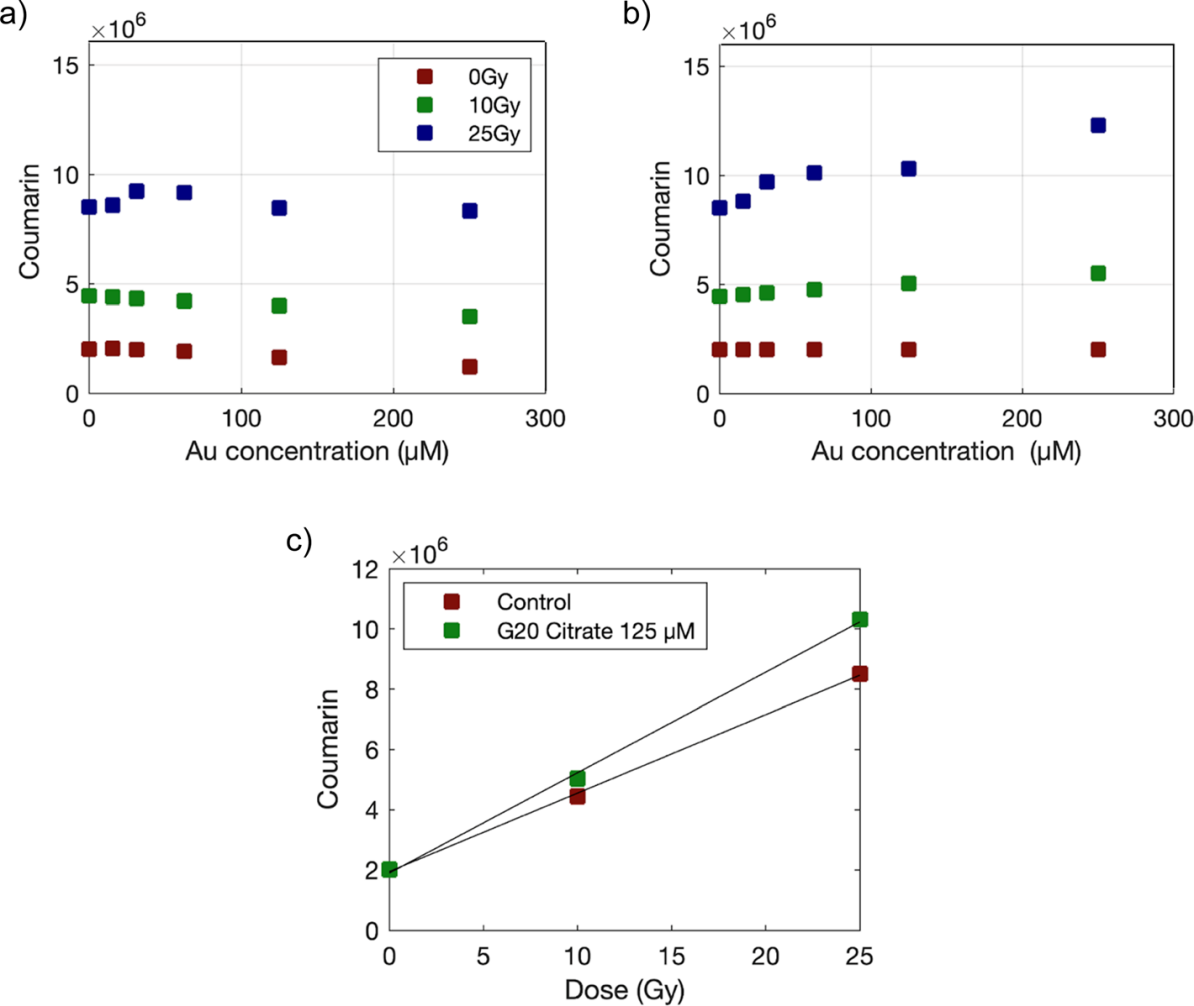
Coumarin fluorescence measurements before (a) and after (b) attenuation correction for samples containing G20 citrate AuNPs, irradiated with γ photons at doses of 0, 10 and 25 Gy. (c) Attenuation corrected fluorescence values for G20 citrate 125 μM Au and control (distilled water) samples plotted against the radiation dose. Data were fitted to straight lines and EF was obtained as the ratio of the slopes obtained for AuNPs and control samples.

**Fig. 3:**
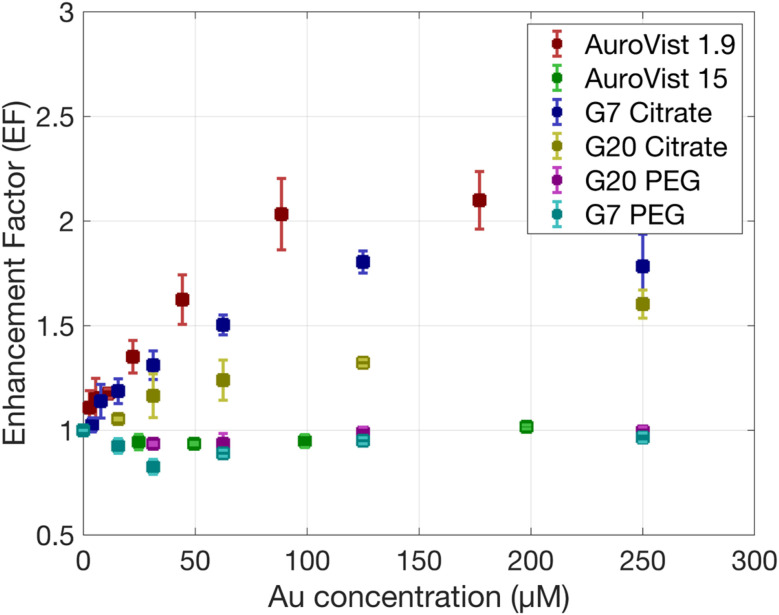
EF obtained for the production of HO· radicals as a function of Au concentration under γ photons irradiation at doses of 0, 10 and 25 Gy for the different AuNPs.

Fig. 2 illustrates the procedure followed to obtain the enhancement factor (EF) that is a measure of how much the presence of AuNPs increases the effectiveness of radiation therapy, in this case γ photon irradiation with AuNPs compared to radiation alone, from the measured coumarin fluorescence values. First, raw fluorescence values attenuated by AuNPs and subjected to increased radiation (ranging from 0 to 25 Gy) show an increase in fluorescence at low Au concentration while values drop at higher concentrations. Attenuation-corrected fluorescence values show a steady increase for higher Au concentrations. We can observe a linear relationship between the fluorescence value and the dose (see Fig. 2c). By performing a linear fit, we can compare the slope obtained for the control and AuNP samples, obtaining the EF. EF values obtained for all AuNPs are shown in Fig. 3. The highest EF was obtained for the smallest AuNPs, AuroVist 1.9 AuNPs, followed by citrate-capped G7 and G20 AuNP. AuroVist 15 and PEG-capped AuNPs did not show any increase in the production of OH·. It can be observed that the EF reach a plateau which, as suggested by Gilles *et al.*^28^ might be due to a competition between HO· scavenging by coumarin and recombination with other radical species generated during water radiolysis.

### 3.3. Radio-activated production of singlet oxygen (^1^O_2_) in the presence of AuNPs

**Fig. 4:**
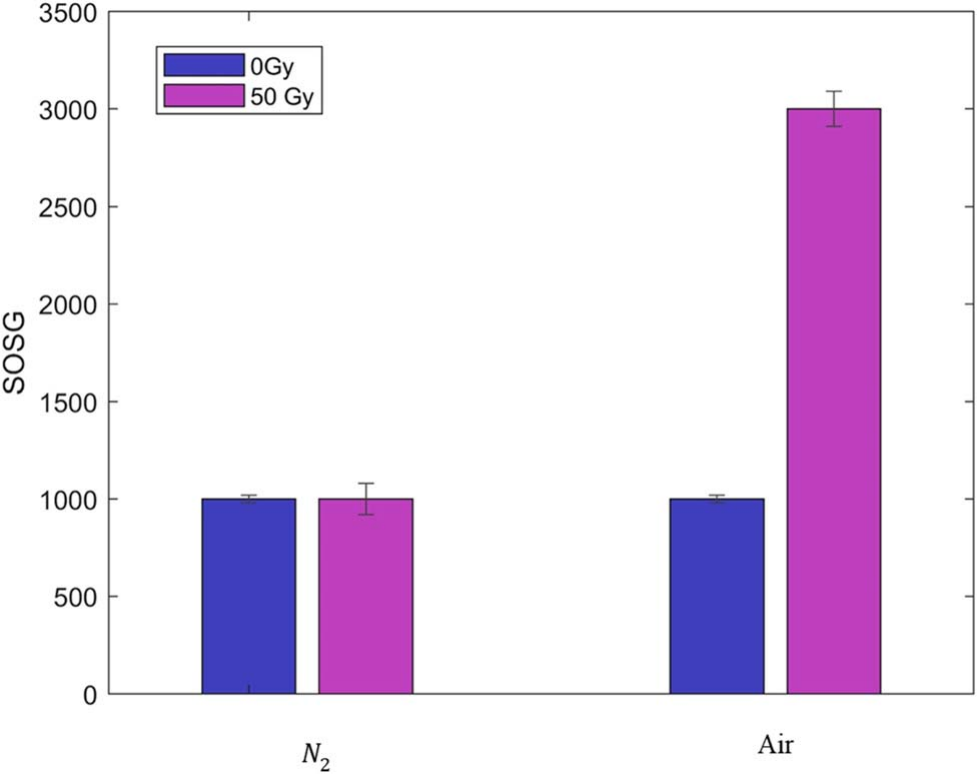
Fluorescence values obtained for SOSG on non-irradiated (0 Gy) and irradiated (50 Gy) samples under air- and nitrogen-saturated conditions.

As mentioned, our intention was to use SOSG probe to monitor the production of ^1^O_2_ but a previous study^29^ concluded that SOSG should not be used with ionizing radiation, as activation was observed in nitrogen saturated samples where no ^1^O_2_ could be produced. However, the authors did not provide evidence that samples remained oxygen-free during the entire experiment. Therefore, we decided to repeat the experiment using an oxygen sensor (PICO-2O, Pyroscience) to study the conditions under which the sample remained oxygen-free throughout both preparation and irradiation processes. Briefly, 1 mL distilled water was introduced in a round bottom flask and sealed with a rubber stopper. The sample was bubbled with nitrogen for 5 minutes and the oxygen concentration was monitored for 30 minutes afterwards obtaining a final concentration below 1%. The same protocol was followed to irradiate SOSG with γ photons, in this case, at 50 Gy on air- and nitrogen-saturated samples. After irradiation, the flasks were opened and fluorescence was measured on 96-well plates. Each condition was repeated three times and the results shown on Fig. 4 reveal that SOSG was only activated in the presence of oxygen. Therefore, we decided to proceed using SOSG as a reliable sensor for the production of ^1^O_2_.

SOSG was only used to monitor ^1^O_2_ production with PEG-coated and commercial AuNPs due to interaction of SOSG with the citrate capping agent.^30^ We confirmed that by incubating G20 citrate AuNPs with SOSG at room temperature for 1 hour and precipitating AuNPs by centrifugation at 3000×*g* for 45 min. The fluorescence at the supernatant decreased as the concentration of AuNP increased (see Fig. S4†), suggesting the adsorption of SOSG to citrate-capped AuNPs.

**Fig. 5:**
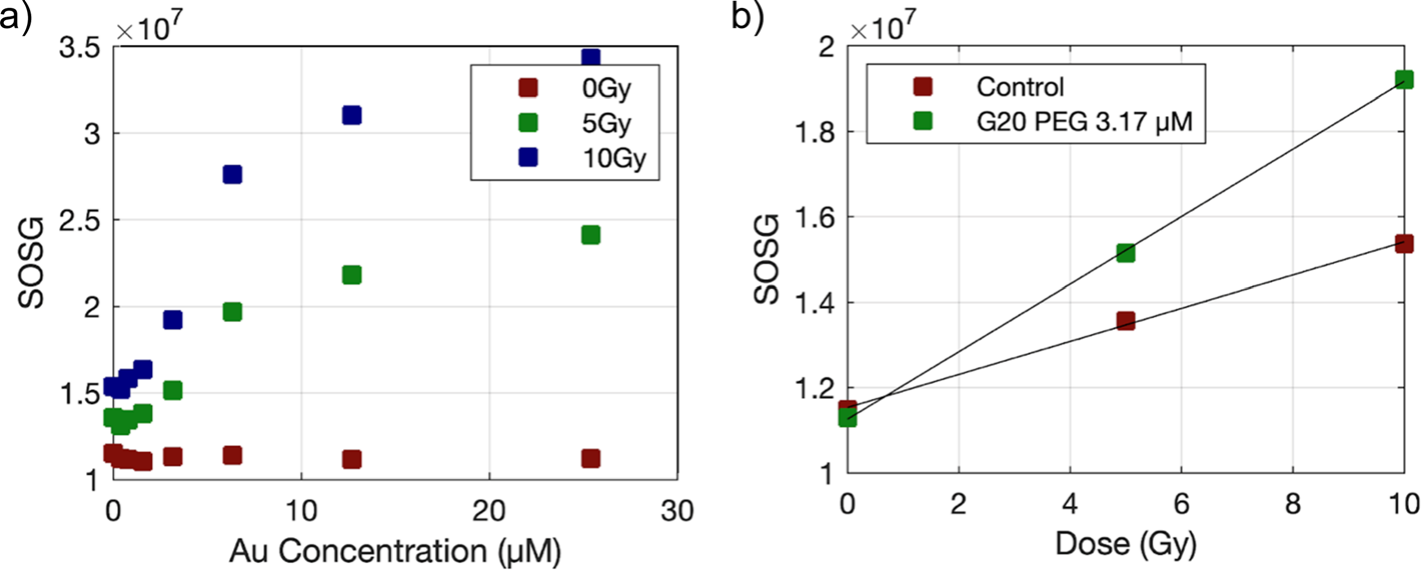
SOSG fluorescence measurements (a) for samples containing G20 citrate AuNPs, irradiated with γ photons at doses of 0, 5 and 10 Gy. (b) Fluorescence values for G20 citrate 6.3 μM Au and control (distilled water) samples plotted against the radiation dose. Data were fitted to straight lines and EF was obtained as the ratio of the slopes obtained for AuNP and control samples.

Fig. 5 shows the raw fluorescent values of SOSG recorded on AuNP samples irradiated with lower doses 0, 5 and 10 Gy. The doses used to irradiate SOSG are lower than those used for coumarin to avoid saturation of fluorescence signals. In this case, no attenuation correction was required due to the low Au concentrations studied. A linear behavior between SOSG values and delivered dose was observed and the slope of the linear fits were further used to determine the EF.

**Fig. 6:**
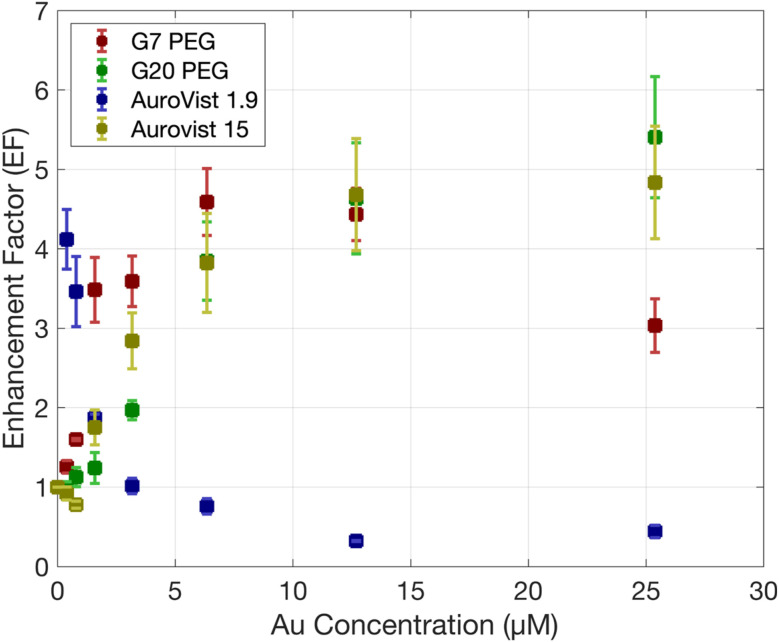
EF of ^1^O_2_ production under γ photon irradiation at doses of 0, 5 and 10 Gy as a function of Au concentration for the different AuNPs.

The EF of ^1^O_2_ production obtained for studied AuNPs is shown on Fig. 6. It can be observed that the EF increases and then rapidly drops for some AuNPs. This effect might be due to a competition between SOSG and the scavenging power of the AuNPs. To verify this, we repeated the irradiation of some AuNPs using a 6-fold increase in SOSG concentration (Fig. S5†), which confirmed a reduction in the saturation of the SOSG signal.

### 3.4. Correlation of EF with AuNP size

**Fig. 7:**
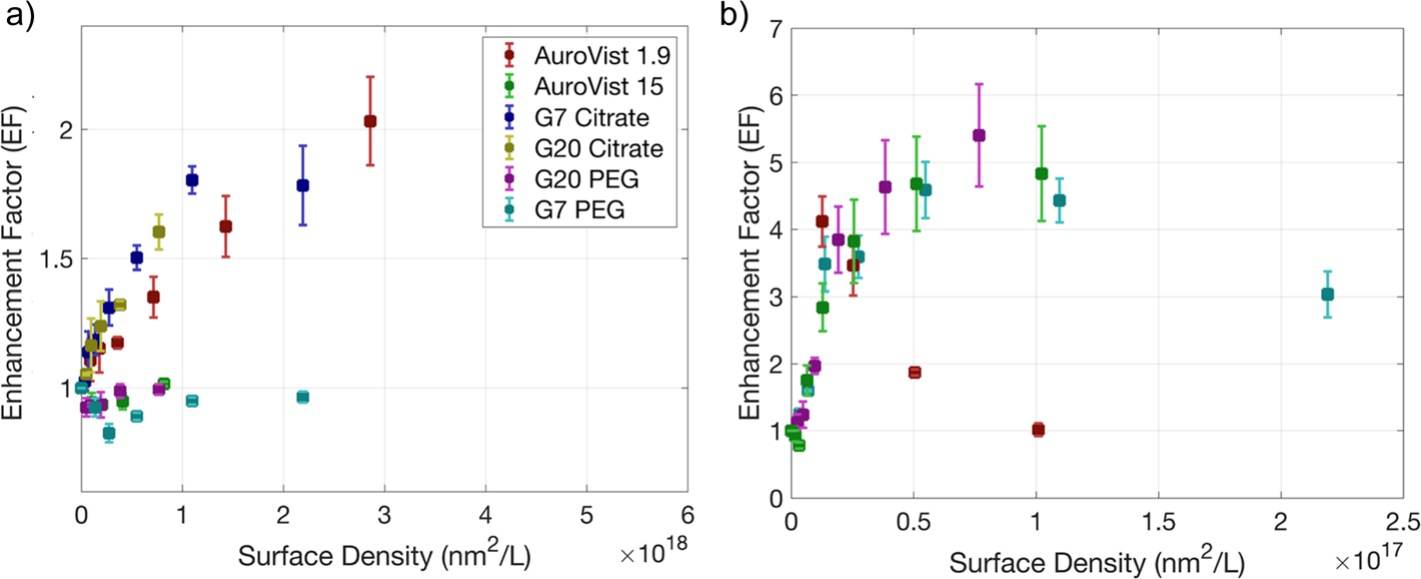
EF of HO· (a) and ^1^O_2_ (b) production under γ photon irradiation at doses of 0, 10 and 25 Gy for HO·, and 0, 5 and 10 Gy for ^1^O_2_, as a function of the AuNP surface density.

The EF obtained for HO· and ^1^O_2_ was represented as a function of the AuNP surface density using the nanoparticle sizes obtained by TEM (see Fig. 7). In this way, a better correlation is shown for most AuNPs suggesting a better relation of EF with the nanoparticle surface area than with Au concentration.

### 3.5. *In vitro* studies

The stability of our AuNPs under experimental cell conditions has been studied by performing UV-Vis spectroscopy measurements of AuNPs dispersed either in water or in DMEM supplemented with 10% FBS after 24 h (see Fig. S6†). For AuroVist 15, the UV-Vis spectra showed stability in both media, with no significant changes in the localized surface plasmon resonance (LSPR) peak. Although the LSPR peak was not clearly visible in AuroVist 1.9 AuNPs, no major changes were observed in the spectra, indicating stability. For G20-PEG and G7-PEG a slight decrease in the intensity of the LSPR peak was observed, which may suggest some minor interaction with serum proteins in DMEM. However, no significant aggregation was apparent. Citrate capped AuNPs (G20-citrate and G7-citrate) suffered a LSPR peak broaden after 24 h in the medium, suggesting partial aggregation and subsequent plasmonic coupling. This behavior is consistent with the known tendency of citrate-coated AuNPs to aggregate in physiological environments due to their interactions with serum proteins.

#### 3.5.1 Cell internalization of AuNPs

The internalization results of AuNPs on MDA-MB-231 cells is shown on Table 2, confirming values between 0.4 and 1.70 pg_Au_ per cell. All the incubations were performed after 24 h and at Au concentration of 200 μM. The internalization of citrate-capped AuNPs could not be properly quantified due to the attachment of AuNPs to the bottom of the well.

**Table 2:** Internalization of AuNPs in MDA-MB-231 cells

AuNP	AuroVist 1.9	AuroVist 15	G7 citrate	G7 PEG	G20 citrate	G20 PEG
Internalization (pg_Au_ per cell)	0.83 ± 0.01	1.70 ± 0.17	—	0.40 ± 0.14	—	0.9 ± 0.3

#### 3.5.2 Toxicity

**Fig. 8:**
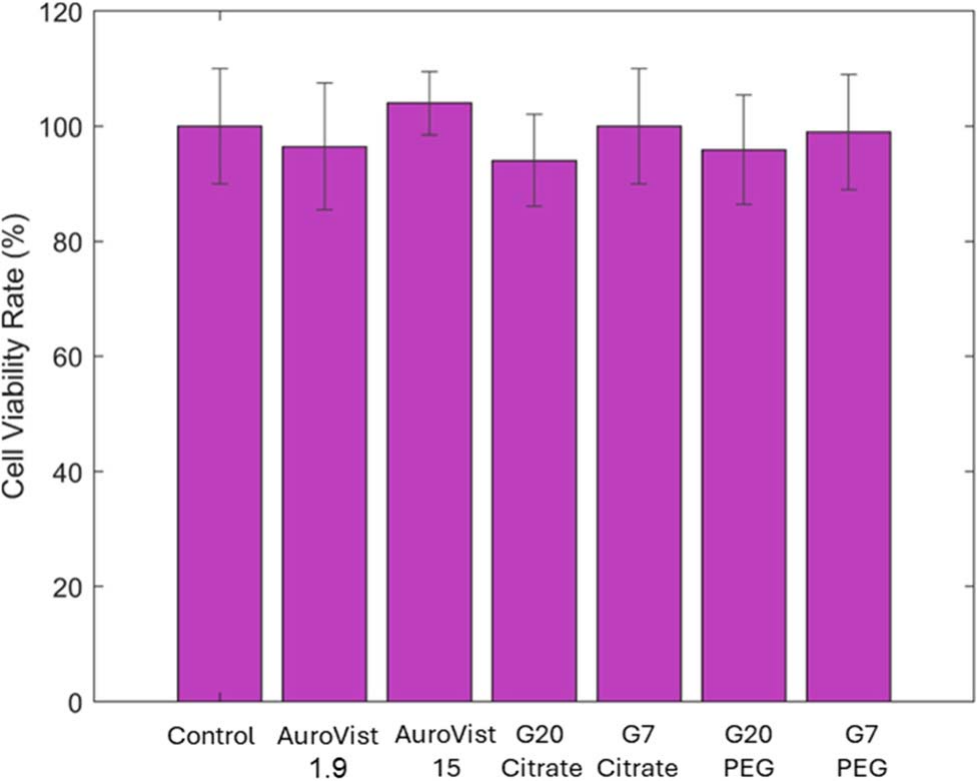
Evaluation of AuNPs toxicity on MDA-MB-231 cells was conducted after 24 hours-treatment with the different AuNPs at 1 mM of Au. The assay was performed using clonogenic tests.

To assess the toxicity of AuNPs on MDA-MB-231 cells, the effect of each AuNP used was studied on cell proliferation *via* the clonogenic assay. As shown in Fig. 8, the 24 hours treatment with any of the assayed AuNPs induced statistically not significant variation on cell proliferation when compared to control cells, demonstrating that none of the AuNPs used are toxic at that concentration.

#### 3.5.3 Radiosensitization effect in cells

**Fig. 9:**
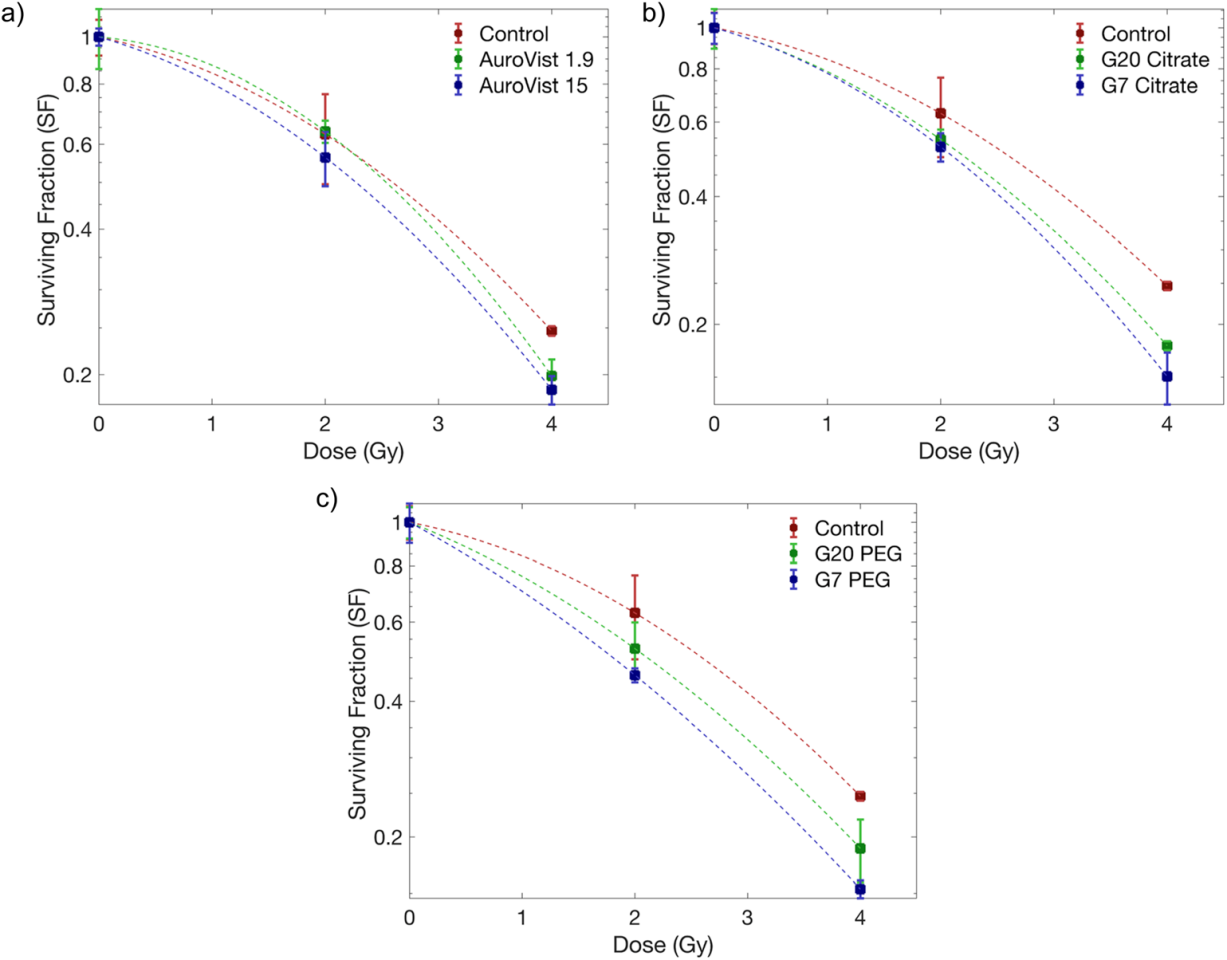
Survival curves for MDA-MB-231 cells incubated with and without (control) AuNPs and irradiated with γ photons. Results are shown for AuroVist 1.9 and AuroVist 15 (a), G20 citrate and G7 citrate (b) and G20 PEG and G7 PEG (c). Dashed lines show the fit to the LQ model.

**Fig. 10:**
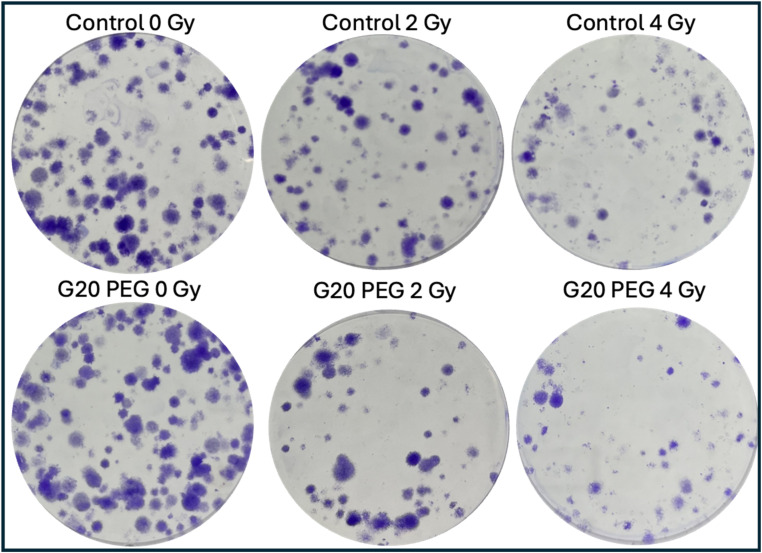
Colony images obtained from clonogenic assays for control and G20 PEG-incubated cells, irradiated with γ photons at doses of 0, 2 and 4 Gy.

The radiation-enhancing effects of the AuNPs were evaluated using a clonogenic survival assay. Fig. 9 shows the survival curves of MDA-MB-231 cells incubated with AuNPs after exposure to γ photons at doses ranging from 0–4 Gy and Fig. 10 shows representative examples of the colony images taken from the clonogenic assays. Results for unlabeled and non exposed control cells are also included. The surviving fractions were normalized to those of unirradiated cells to account for the direct cytotoxic effect of the AuNPs. Quantitative results obtained from survival curves are shown on Table 3.

**Table 3:** Quantitative analysis of survival curves of MDA-MB-231 cells incubated with and without (control) AuNPs and irradiated with gamma photons. Results are shown for the parameters obtained from the fit to the LQ model (*α*, *β*, and *α*/*β*), the surviving fraction at 4 Gy (SF_4_) and the sensitizer enhancement ratio (SER). *p* values for SF_4_ are also shown

AuNP	*α* [Gy^−1^]	*β* [Gy^−2^]	*α*/*β* [Gy]	SF_4_	*p*	SER
Control	0.110 [−0.105, 0.331]	0.059 [−0.015, 0.134]	1.9	0.246 ± 0.006	—	1
AuroVist 1.9	0.046 [−0.170, 0.261]	0.090 [0.010, 0.169]	0.5	0.199 ± 0.016	0.0087	1.01
AuroVist 15	0.154 [0.017, 0.290]	0.067 [0.017, 0.116]	2.3	0.186 ± 0.012	0.0015	1.10
G20 citrate	0.174 [−0.013, 0.362]	0.064 [−0.004, 0.132]	2.7	0.178 ± 0.004	<0.0001	1.12
G7 citrate	0.173 [−0.003, 0.348]	0.075 [0.009, 0.142]	2.3	0.151 ± 0.020	0.0016	1.16
G20 PEG	0.230 [0.036, 0.423]	0.047 [−0.021, 0.115]	4.9	0.189 ± 0.030	0.0303	1.15
G7 PEG	0.314 [0.111, 0.516]	0.039 [−0.034, 0.112]	8.1	0.153 ± 0.007	<0.0001	1.27

An increase in *α* values for most AuNP-incubated cells suggests a rise in direct cellular damage due to the presence of AuNPs within the cells during irradiation. In all cases, SF_4_ values show significant differences between cells incubated with AuNPs and control cells.

#### 3.5.4 Intracellular ROS quantification

**Fig. 11:**
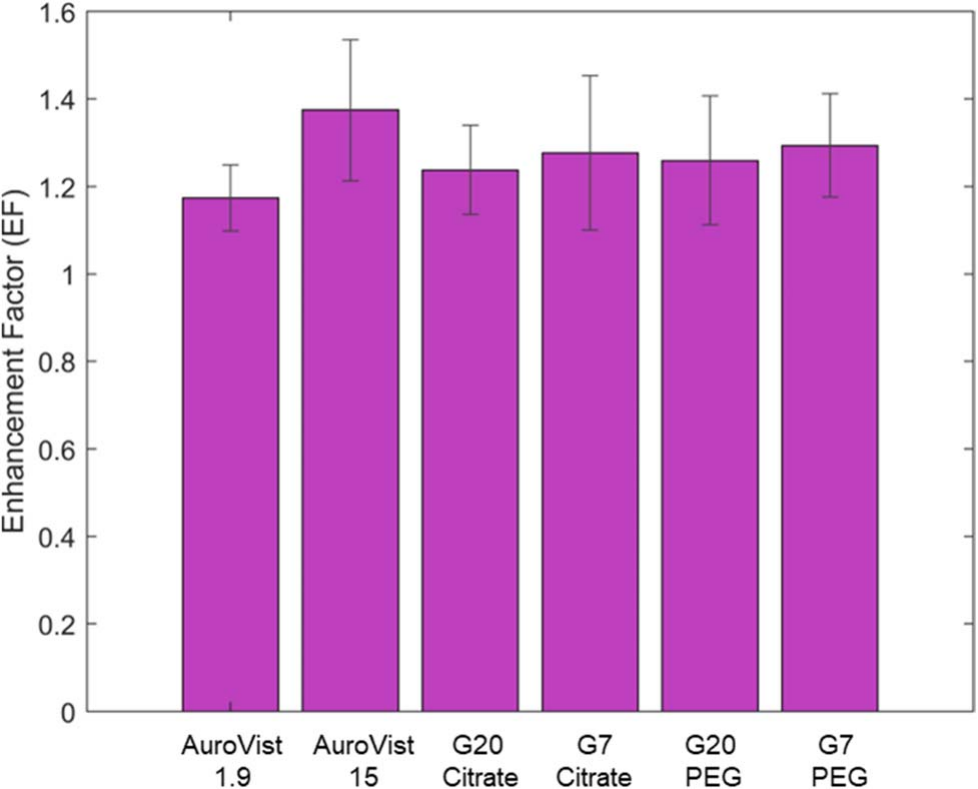
EF of intracellular ROS production under γ photon irradiation at doses of 0, 5 and 10 Gy.

The intracellular ROS production was quantified by incubating cells with AuNPs, treating them with DCFH-DA, and measuring fluorescence intensity after photon irradiation at doses of 0, 5, and 10 Gy. The EF for intracellular ROS production under photon irradiation was analyzed for various types of AuNPs. As shown in Fig. 11, no significant differences were observed in EF values for intracellular ROS production among the different nanoparticle formulations, although AuroVist 1.9 exhibited a slightly lower enhancement factor. This observation aligns with the lower *α* parameter reported in the LQ model for this AuNPs, which is associated with reduced direct damage under the tested conditions. It is important to note that DCFH-DA measures total ROS production without differentiating between specific species. This could explain why the differences observed in specific ROS species, such as OH· and ^1^O_2_, as previously shown in this work, do not translate into significant variations in the overall intracellular context.

## 4. Discussion

In this study, we evaluated the chemical and biological radiosensitizing effects of AuNPs with different sizes and coatings in order to better understand the mechanisms through which AuNPs enhance radiation therapy. The goal was to gain deeper insights into the mechanisms by which AuNPs enhance the effectiveness of radiation therapy. Although the mechanisms are mostly known, research continues to refine their size, coating, and targeting to enhance their radiosensitizing potential and maximize their therapeutic effect.

First, we quantified the production of HO· and ^1^O_2_ using fluorescence probes. Our results demonstrated that the highest EF for HO· production was achieved with AuroVist 1.9 nm AuNPs, followed by citrate-capped G7 and G20 AuNPs. This confirms the size dependence previously reported by others,^13,14^ which was also confirmed for the production of ^1^O_2_ in very small NPs with a high surface-to-volume ratio. However, PEG-capped AuNPs did not show a significant enhancement in HO· generation, suggesting that the PEG coating may hinder the chemical enhancement effect as also shown in previous studies.^18^ Interestingly, PEG-capped AuNPs exhibited a large EF for ^1^O_2_ production, indicating that multiple reactive species can contribute to the radiosensitizing effect of AuNPs. Therefore, it is essential to fully characterize these reactive species to obtain a comprehensive understanding of the response of AuNPs to irradiation. No results for ^1^O_2_ production could be obtained for citrate-capped AuNPs since SOSG was adsorbed to the surface of the nanoparticle.

On the other hand, clonogenic assays revealed that the radiosensitization effect of PEG-capped AuNPs was stronger than that of their citrate-capped counterparts. These results suggest that, even though PEG-capped AuNPs do not show enhancement on the production of OH·, they still retain radiosensitizing capabilities, potentially related to the enhanced ^1^O_2_ production encountered in this study. According to particle size, smaller citrate- and PEG-capped AuNPs showed larger radiosensitizing effect than their larger counterparts in agreement with what was observed for ROS production. In the case of AuroVist AuNPs, the correlation between chemical and biological results is less clear. It is to note that their capping agent is unknown, and differences in cell internalization and localization should also be considered. We included AuroVist AuNPs in our study as several previous studies included the same nanoparticles as radiosensitizers^27,31,32^ although irradiation conditions were not identical in all cases. A previous study also showed the size-dependent radiosensitization effect of PEG-coated AuNPs.^33^ In that study, AuNPs with PEG 5000 at concentrations of 0.05–0.1 mM were tested in HeLa cells, whereas this study utilized PEG 2000-coated AuNPs at 1 mM in MDA-MB-231 cells. These differences in PEG coating, concentration, and cell lines may account for the observed variations in radiosensitization and toxicity. Zhang *et al.* reported higher toxicity due to PEG-cell interactions, which contrasts with the findings of this study.

Further studies are needed to better understand the relation between ROS production, nanoparticle characteristics, and biological responses. An optimization of nanoparticle coatings must be performed in order to balance stability and radiosensitizing efficacy. As suggested by Yogo *et al.*,^34^ exploring the impact of nanoparticle surface charge on radiosensitization could offer additional insights. While our study focused exclusively on negatively charged AuNPs, future research could investigate positively charged AuNPs to determine their influence on radiosensitization and ROS production. By addressing these areas, we can refine the application of nano-radiosensitizers and move closer to achieving the goal of maximizing therapeutic efficacy while minimizing collateral damage in radiation therapy.

## 5. Conclusions

In this study, we evaluated the chemical and biological radiosensitizing effects of AuNPs with different sizes and coatings to better understand the mechanisms through which AuNPs enhance radiation therapy. Our findings demonstrated a significant size dependence in the production of HO· and ^1^O_2_. Interestingly, PEG-capped AuNPs did not enhance HO· production but a large EF for ^1^O_2_ production was obtained, indicating the contribution of multiple reactive species to the radiosensitizing effect. Clonogenic assays further revealed that PEG-capped AuNPs had stronger radiosensitizing effects compared to citrate-capped AuNPs and that smaller AuNPs provide a larger biological effect, consistent with the observed ROS generation. Our study underscores the necessity to perform both chemical and biological studies to fully understand the radiosensitizing efficacy of AuNPs. Chemical studies are essential for understanding how AuNPs produce reactive species and interact with radiation. On the other hand, biological studies are necessary to assess how these mechanisms translate into effective radiosensitization at the cellular level. Future research should focus on these aspects to refine nano-radiosensitizer applications, aiming to maximize therapeutic efficacy, while minimizing collateral damage in radiation therapy.

## Data availability

The datasets used and/or analyzed during the current study are available from the corresponding author upon reasonable request.

## Author contributions

Esther Loscertales: investigation, formal analysis, writing – original draft, writing – review & editing. Rosalía López-Méndez: investigation, formal analysis, writing – review & editing. Jesús Mateo: methodology, investigation, writing – review & editing. Luis Mario Fraile: methodology, investigation, writing – review & editing. José Manuel Udías: methodology, investigation, writing – review & editing. Ana Espinosa: conceptualization, supervision, methodology, funding acquisition, writing – review & editing. Samuel España: conceptualization, supervision, methodology, funding acquisition, writing – review & editing, writing – original draft.

## Conflicts of interest

The authors declare no competing financial interest.

## Supplementary Material

NA-OLF-D4NA00773E-s001
