# Supplementary material for: Impact of gold nanoparticle size and coating on radiosensitization and generation of reactive oxygen species in cancer therapy
Source: Nanoscale Adv. 2025 Jan 9;7(4):1204–14. doi: 10.1039/d4na00773e (PMC11712209; doi:10.1039/d4na00773e)
Supplement: NA-007-D4NA00773E-s001 [file NA-007-D4NA00773E-s001.pdf]

Supplementary Information (SI) for Nanoscale Advances.
This journal is © The Royal Society of Chemistry 2024

SUPPLEMENTARY DATA

TITLE

Impact of gold nanoparticle size and coating on radiosensitization and generation of reactive oxygen species in cancer therapy

AUTHORS

E. Loscertales[a,b], R. López-Méndez[c], J. Mateo[d], L. M. Fraile[a,e], J. M. Udias[a,e], A. Espinosa[f,c], S. España[a,b,d]

[a] Grupo de Física Nuclear, EMFTEL & IPARCOS, Universidad Complutense de Madrid, Pl. de las Ciencias, 1, Moncloa-Aravaca, Madrid, Spain
[b] Instituto de Tecnologías Físicas y de la Información "Leonardo Torres Quevedo" (ITEFI-CSIC), C/Serrano, 144, Madrid, Spain
[c] IMDEA Nanociencia, C. Faraday, 9, Fuencarral-El Pardo, Madrid, Spain
[d] Centro Nacional de Investigaciones Cardiovasculares (CNIC), C. de Melchor Fernández Almagro, 3, Fuencarral-El Pardo, Madrid, Spain
[e] Instituto de Investigación del Hospital Clínico San Carlos (IdISSC), C. del Prof Martín lagos, S/N, Moncloa-Aravaca, Madrid, Spain
[f] Instituto de Ciencia de Materiales de Madrid (ICMM-CSIC), Campus de Cantoblanco, C. Sor Juana Iés de la Cruz, 3, Fuencarral-El Pardo, Madrid, Spain

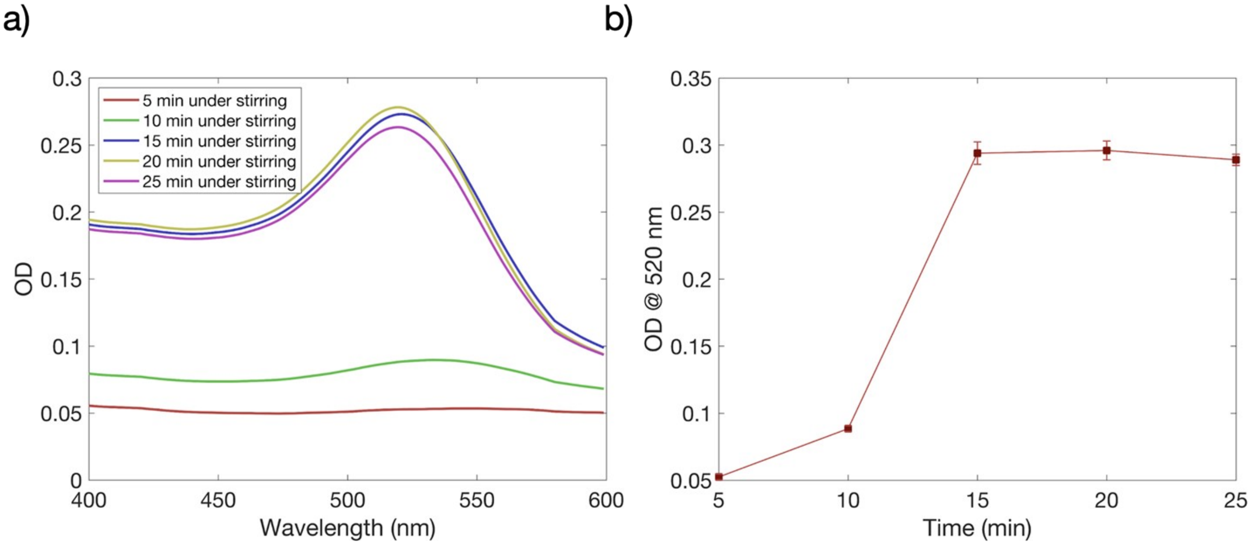

Figure S1. The growth of G20 citrate AuNPs was monitored by recording their optical density at different reaction times. Panel (a) shows the UV-vis spectra and panel (b) shows the increase of the OD@520nm with the reaction time.

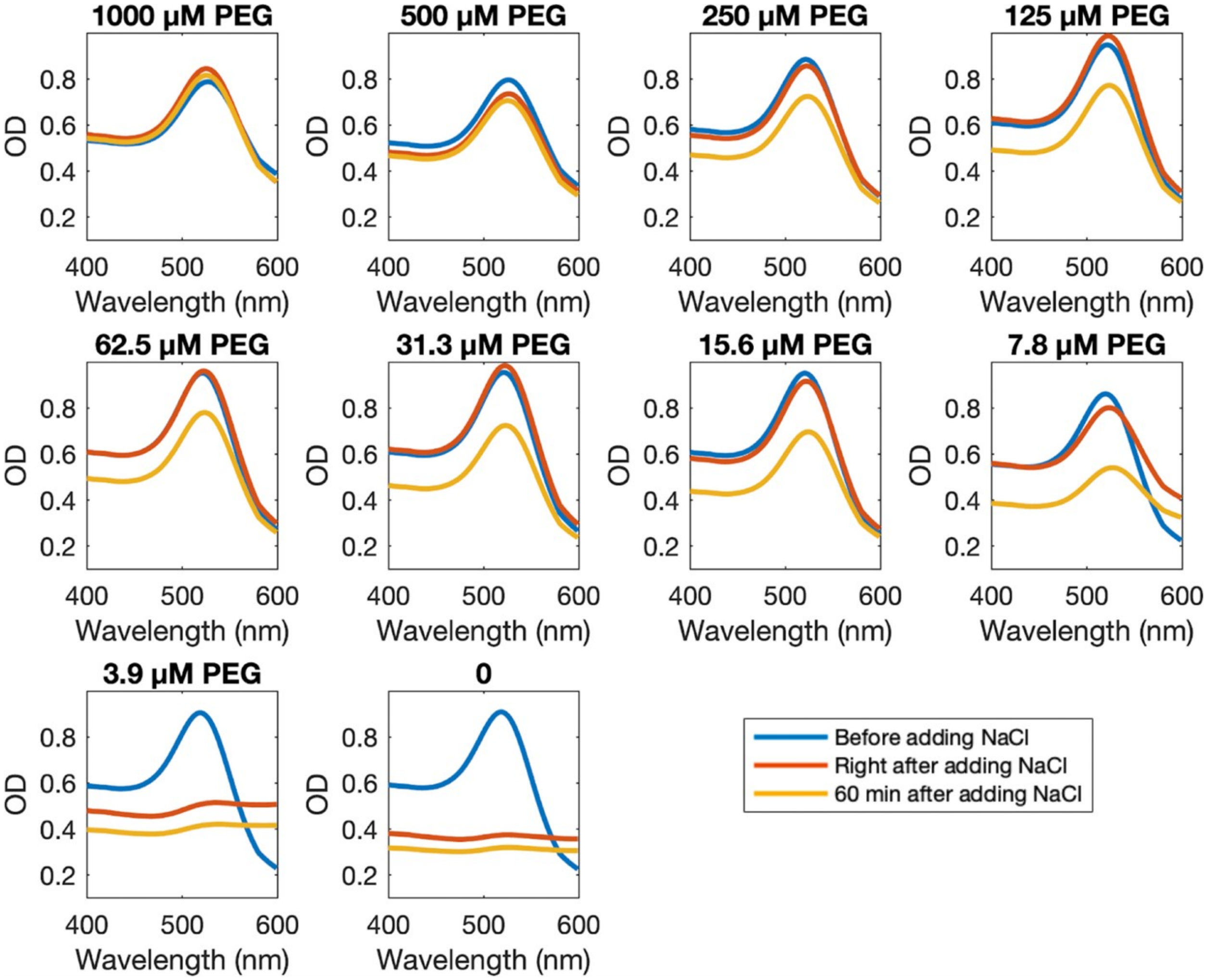

Figure S2. G20 Citrate AuNPs incubated with PEG at different concentrations before, right after and 60 minutes after adding NaCl (250 μM).

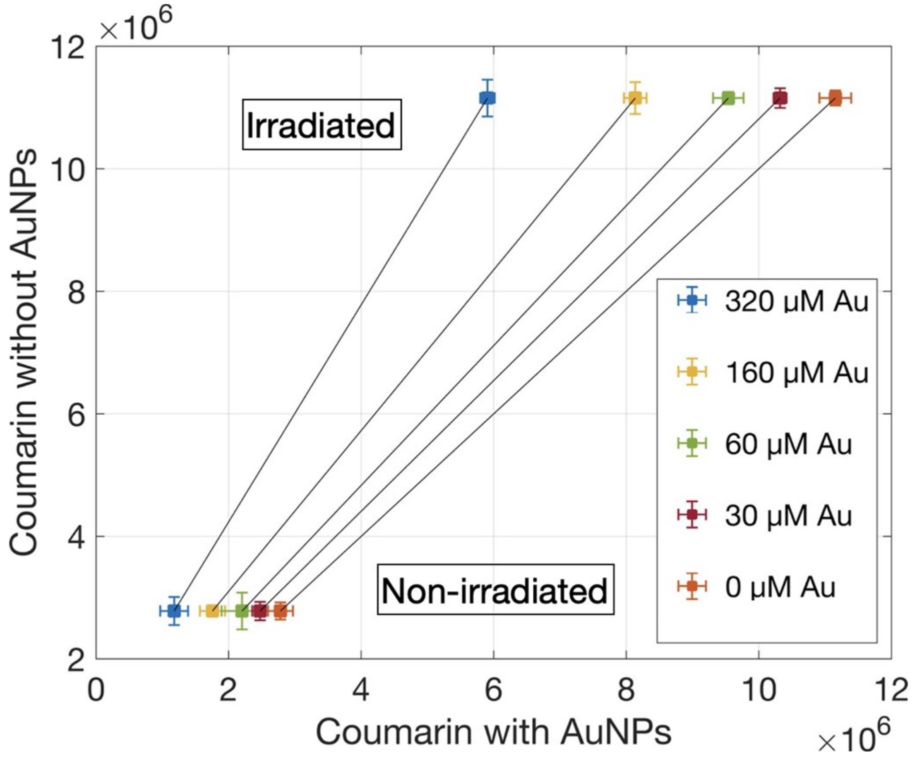

Figure S3. Calibration curves obtained for the attenuation of coumarin fluorescence by G20 Citrate AuNPs at different concentrations (30 - 320 μM Au). Fluorescent values of irradiated and non-irradiated coumarin are compared for samples with and without AuNPs.

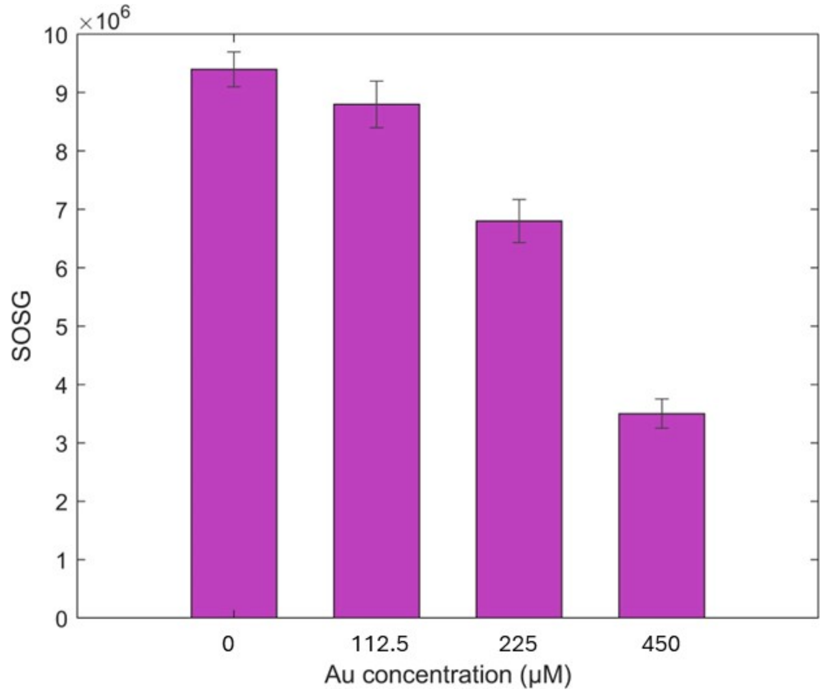

Figure S4. SOSG fluorescence was measured at the supernatant after mixing it with different concentrations of G20 Citrate AuNPs during 1 hour and centrifuge the mixture for 45 minutes. Values drop for higher Au concentration due to adsorption of SOSG to citrate capped AuNPs.

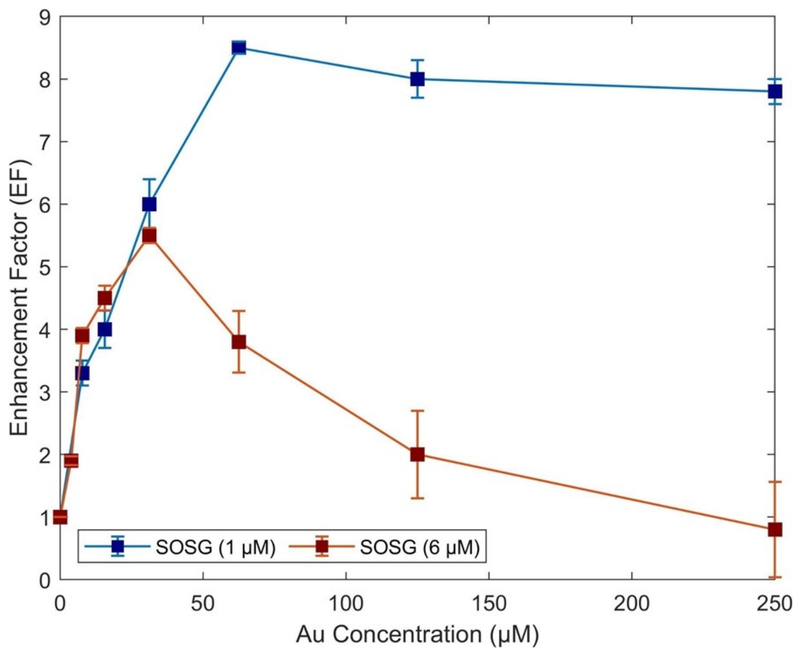

Figure S5. EF of $^1O_2$ for G20 PEG AuNPs under γ photon irradiation as a function of Au concentration when SOSG concentration was 1 or 6 µM.

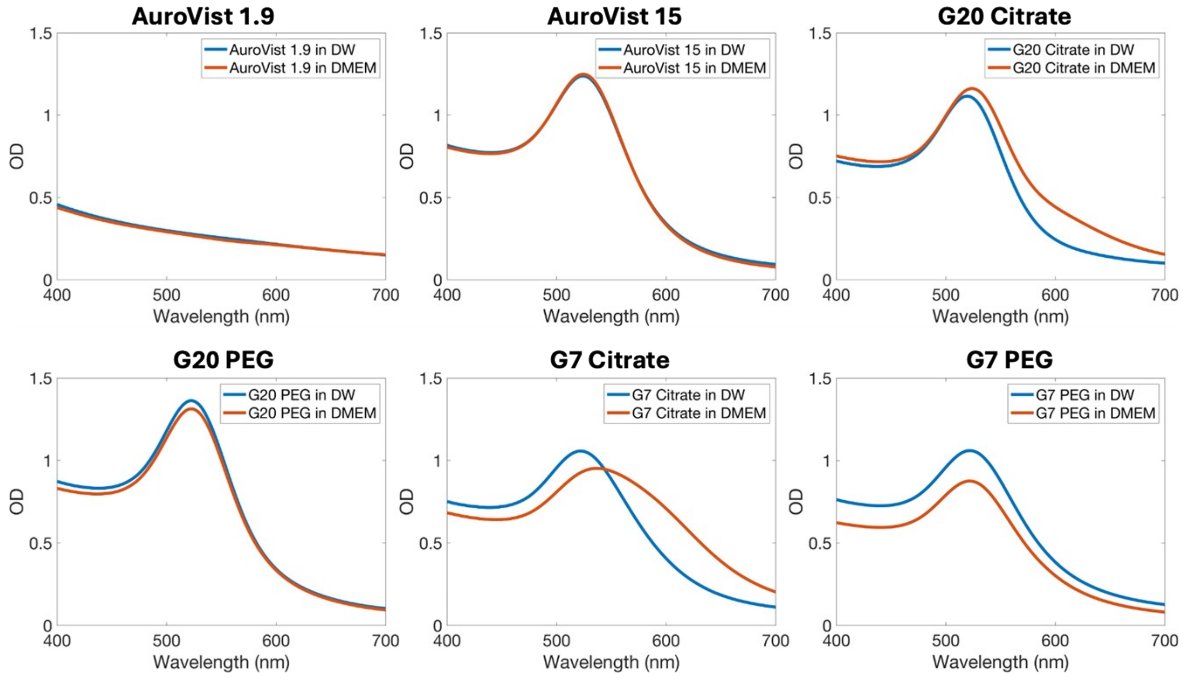

Figure S6. UV-Vis spectroscopy measurements of AuNPs after 24 h of incubation dispersed either in water or in DMEM supplemented with 10% FBS to check their stability.
